# The Design and Use of an Optical Mapping System for the Study of Intracardiac Electrical Signaling

**DOI:** 10.1016/s0972-6292(16)30521-6

**Published:** 2012-07-28

**Authors:** Maneesh Shrivastav, Megan B Ghai, Ashish Singal, Paul A Iaizzo

**Affiliations:** 1 University of Minnesota, Dept of Biomedical Engineering & Surgery; 2 University of Minnesota, Dept of Surgery; 3 Medtronic, India

**Keywords:** optical mapping, cardiac mapping, signal processing, voltage sensitive dye

## Abstract

Fluorescent optical mapping of electrically active cardiac tissues provides a unique method to examine the excitation wave dynamics of underlying action potentials. Such mapping can be viewed as a bridge between cellular level and organ systems physiology, e.g., by facilitating the development of advanced theoretical concepts of arrhythmia. We present the design and use of a high-speed, high-resolution optical mapping system composed entirely of "off the shelf" components. The electrical design integrates a 256 element photodiode array with a 16 bit data acquisition system. Proper grounding and shielding at various stages of the design reduce electromagnetic interference. Our mechanical design provides flexibility in terms of mounting positions and applications (use for whole heart or tissue preparations), while maintaining precise alignment between all optical components. The system software incorporates a user friendly graphical user interface, e.g., spatially recorded action potentials can be represented as intensity graphs or in strip chart format. Thus, this system is capable of displaying cardiac action potentials with high spatiotemporal resolution. Results from cardiac action potential mapping with intact mouse hearts are provided. It should be noted that this system could be readily configured to study isolated myocardial biopsies (e.g., isolated ventricular trabeculae). We describe the details of a versatile, user-friendly system that could be employed for a magnitude of study protocols.

## Introduction

The study of excitation waves can provide insightful information relative to the spatial and temporal characteristics of healthy and/or diseased cardiac tissue. In other words, by recording at the cellular level, the initiation and/or propagation of action potentials can provide valuable information regarding the genesis and mechanisms of arrhythmias.

Contact electrode techniques of recording single monophasic action potential probes or multielectrode arrays are a common way to investigate the distribution of action potentials [1]. Though surface and contact electrodes can provide an indication of the spread of excitation and repolarization, the interpretation of such data can be very complex and/or difficult in some circumstances, e.g., after a defibrillation shock or during the study of spatiotemporal organization of repolarization in arrhythmogenesis [2]. Furthermore, the physical contact of these electrodes on the tissue can alter electrical and physical characteristics of the tissue to be examined. In contrast, noncontact optical mapping involves the use of specialized imaging techniques and cellular treatments of voltage sensitive dyes. During the last two decades, the development, use, and optimization of such multisite fluorescence measurements with high temporal and spatial resolution (optical mapping) has enabled the visualization of wavefronts in Langendorff-perfused whole heart models, isolated tissues biopsies, and within microscopic preparations of cardiomyocyte monolayers (cell cultures) [3-5].

This paper describes the design and use of an optical mapping system for the investigation of cardiac electrical activity from intact rat hearts or isolated tissue samples. These techniques employ a signal acquisition system with a 256 channel photodiode array and use of voltage sensitive dye, di-4-ANEPPS. Our system is user-friendly and provides high temporal and spatial resolution, a flexible dynamic range, and variable sampling frequency. Unlike the many high quality but expensive optical mapping systems currently used in many labs, our system was designed with readily available components, avoiding the cost of complex hardware and software customization. For example, the use of such a system has significance for facilitating the analyses of action potential wavefront propagations during arrhythmia in normal or diseased cardiac tissues, in a robust and cost efficient manner.

## Methods

All procedures described in these experiments were approved by the University of Minnesota Animal Care and Use Committee, and animal care was in accordance with the National Institutes of Health (NIH) Guidelines for the Care and Use of Laboratory Animals.

### Animal model and whole heart preparation

Adult Sprague-Dawley rats (240.0 g ± 10%) were used for these experiments. Rats were visually inspected, weighed, and injected with 300 units/kg of heparin 10 minutes before surgery, to prevent blood coagulation and myocardial ischemia or infarction during surgical explantation. Rats were then placed in an induction chamber with the oxygen flowmeter adjusted between 0.8 to 1.5 L/min. Deep general anesthesia was induced via administration of inhalant anesthetic isoflurane by adjusting the vaporizer to 2-4% until the reflexes were suppressed.

To harvest hearts, each animal was stretched out in a supine position on the dissection platform. A midline incision was made through the abdominal wall musculature near the lower edge of the sternum (xiphoid process). A medial sternotomy was performed, the great vessels were dissected, and the heart was removed. Upon explant, the heart was perfused with and immersed in a cold cardioplegic solution (4ºC). While arrested, the heart was cannulated using a tissue clip, followed by silk sutures (Ethicon, Somerville, NJ, USA). Care was taken to prevent air bubbles from entering the cannula. The hearts were then transferred from the extraction site to the mapping lab (approximately 30-40 minutes).

To reinitiate electrical activity within the hearts, Krebs solution was perfused continuously while gradually increasing the temperature of the solution to 30ºC. The Krebs solution had the following composition (mM):NaCl (118.0), D-mannitol (16.0), D-glucose (11.5), NaHCO_3_ (20.0), 2Na-EDTA·2H_2_O (0.32), KCl (4.5), MgCl_2_·6H_2_O (1.46), NaH_2_PO_4_·H_2_O (1.2), CaCl_2_·2H_2_O (1.81), and insulin (10 u/l).

Next, the transmembrane voltage sensitive fluorescent dye, di-4-ANEPPS (Molecular Probes, Portland, OR, USA), was loaded into the myocardial cells to detect time-dependent cell membrane potential changes. This dye had a maximum absorption near 475 nm and a maximum emission around 617 nm [6]; the dye became strongly fluorescent upon binding to cell membranes [7]. A stock solution of 8 mM/L was created by dissolution of 5 mg of di-4-ANEPPS in 1.3 ml of dimethyl sulfoxide (DMSO). The dye was added to the superfusate 3-5 minutes prior to imaging. Care was taken to maintain the temperature of the dye close to that of the Krebs solution, and to manually perfuse the dye slowly to avoid acute insult to the tissue. It should be noted that in using such an approach, the depth of penetration of the dye into the preparation was a subject of debate, though studies by other researchers have predicted penetration depths between 144-500 µm [7]. The dye was recirculated back into the main perfusion chamber via a Micropore filtration apparatus.

### Perfusion apparatus

**Figure 1:**
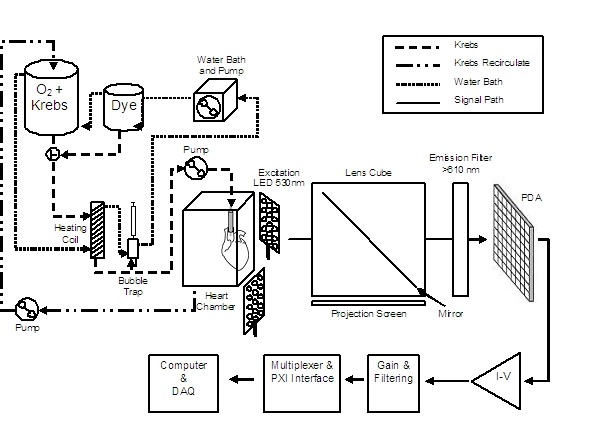
Block diagram view of the mapping system hardware. PDA= photodiode array; DAQ=data acquisition boards.

A temperature, oxygen, and flow-controlled perfusion system was used to maintain tissue viability by infusing Krebs and voltage sensitive dye into the cannulated aorta at a controlled rate of approximately 2 ml/min. The perfusion system consisted of a 500 mL chamber for Krebs and dye (Radnoti Glass Technology Inc., Monrovia, CA, USA) and a heated circulating water bath (Polystat, Cole-Parmer Instrument Company, Vernon Hills, IL, USA). The solution was routed through: a filter to trap any particles or residue, a heating coil (Radnoti) to maintain the temperature of perfusate, a bubble trap (Radnoti) to eliminate microbubbles that might cause ischemia or infarction, and finally to the aortic cannula (Radnoti), as shown in Figure 1.

The entire perfusion system was maintained at a constant temperature by continuously circulating temperature-controlled water via the circulating water bath around the outer water jacket of all glass components. Depending on the environmental conditions (ambient temperature, etc.), the water bath was maintained at a constant temperature of approximately 45ºC, such that the temperature of the perfusate entering the heart was approximately 35ºC. The isolated rat hearts spontaneously and rhythmically contracted when the temperature in the chamber rose to between 29-31ºC. Next, each heart was positioned against the imaging window with the help of pistons built into the heart chamber, such that the majority of the left ventricular epicardial surface was within the mapping field. Yet, to avoid temperature gradients, the hearts were immersed in the coronary effluent. A recirculating pump (Permco, Streesboro, OH, USA) was used to pump the coronary effluent back into the Krebs solution chamber for reoxygenation, rewarming, and perfusion into the tissue preparation. This process also assisted in exploiting the use of di-4-ANEPPS that was washed out during perfusion.

### Hardware

The hardware for this optical mapping system was composed of: 1) the mechanical apparatus (optical lenses and filters, mechanical fixtures, illumination, vibration isolation, etc.); 2) the computer hardware (photodiode array, CPU, CCD camera, etc.); 3) an optical system (LED source, lens assembly with mirrors, filters, and projection screen, and isolation table); and 4) the software system (graphical user interface, digital filtering implementation).

### Data acquisition and computer hardware

**Figure 2:**
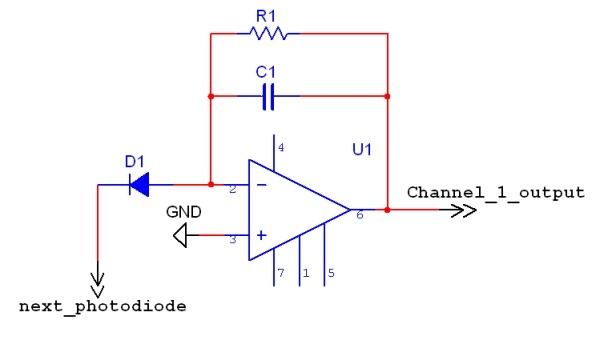
Block diagram of the current to voltage amplifier. Light entering the photodiode, D1, is converted to voltage via the feedback resistor. The photodetector has 256 feedback circuits. R1=100 MΩ and C1=1 pF.

A two-dimensional 256 element photodiode array (model K29-B60026-C4675-102, Hamamatsu Photonics, Hamamatsu City, Japan) was used as the main imaging hardware. The photodiode array (PDA) provided a spectral response of 400-1000 nm, an amplifier gain of 108 volts/amp, and an active viewing area of 17.45 mm x 17.45 mm (1.1 mm x 1.1 mm per element with 0.15 mm between diodes). The PDA operated by converting photocurrent (light energy) into a voltage by a feedback circuit as shown in Figure 2. A dual output DC power supply provided ±15 V directly to the PDA (Model 3620A, Agilent, Santa Clara, CA, USA). A tri-core shielded cable (1 m; Hitachi Cable Ltd., Tokyo, Japan) was used to provide positive, negative, and ground signals to the PDA. The shield of the cable was specifically soldered to the equipment ground system.

The elements of the PDA were connected to four data acquisition (DAQ) boards (Model PXI-6225, National Instruments, Austin, TX, USA). Each board received 64 analog inputs from the PDA. The 16 bit DAQ boards were configured in a non-referenced, single-ended mode. The reference signal was provided by the ground pins on the PDA. It should be noted that this reference signal was isolated from the ground signal used for grounding power cables, as both signals should be treated separately.

Ensuring a favorable signal-to-noise ratio for the overall system required stable and electromagnetically clean connections from the PDA to the DAQ boards. Each of the four DAQ boards had two high-density connectors requiring an interface of eight total connections among the 256 PDA elements. To connect the PDA with the boards, eight shielded I/O connector boxes were used (Model SCB-68, National Instruments); the lids of the boxes were removed. The boxes were connected in pairs by installing a hinge joint such that the boxes could be opened for wiring and closed when in use. This configuration also allowed one pair of boxes to be matched with one DAQ board. Sheet metal side panels were created to seal any open areas; these panels were connected to the ground signal.

Importantly, the breakout boxes had screw-in terminals that facilitated connections from the PDA and high-density EPM style connectors that enabled stable connections with the DAQ boards via a 1 m cable that featured individually twisted cable pairs for noise reduction (Model SHC68-68, National Instruments). Between the PDA and the breakout boxes, 80 conductor shielded cables (1 m, 28 gauge; model 3600G/80, 3M Inc., Minneapolis, MN, USA) were used to connect each element of the PDA. The shield of each cable was soldered to the ground output of the DC power supply; thus, this cable configuration provided 35 db average shielding effectiveness.

The five PXI style boards (four DAQ boards and one image acquisition board) were housed in a general purpose eight-slot chassis (model PXI-1042, National Instruments) that provided power, ground, forced air cooling, and electrical isolation to each card. PC control of the PXI boards was provided by a multisystem extension interface, high-bandwidth copper cable link (model PXI-8331, National Instruments).

Data were acquired with a Pentium IV computer (with hyperthreading) with a processor speed of 3.4 Ghz and 3.0 GB of internal 533 MHz RAM (model GX620, Dell Inc., Round Rock, TX, USA). Hyperthreading aids in the multitasking of applications and decreases the number of dependent instructions on the pipeline.

### Mechanical apparatus

The optics in front of the PDA elements included two light filters with a cube fixture to house the optics, a removable mirror for viewing the tissue preparation on a permanently attached frosted glass projection screen, and a compound lens for focusing on the preparation in the specimen dish. The filter specifications were chosen according to the emission and excitation characteristics of di-4-ANEPPS. The emission filter passed wavelengths of >610 nm. The filters were placed in a bean splitter cube (Newport Oriel Instruments, Irvine, CA, USA); the cube was modified to hold a frosted glass filter at a fixed distance from the edge of the cube. This distance was equivalent to the distance from the cube to the PDA surface. When a mirror was inserted in the cube, the frosted glass served as a projection screen on which the image of the illuminated preparation appeared. The mirror deflected the light from the preparation onto the screen with a 1:1 scale to the photodiode surface. This image was then captured with a digital camera. This setup allowed for: 1) visualization of exactly the same area that the PDA was imaging; and 2) manual focusing on the preparation. A compound lens was attached to the cube using a camera lens mount (model CLM-N, Newport). The 50 mm 1.4f lens (Nikon USA, Melville, NY, USA) provided adequate focal length for this experiment.

**Figure 3:**
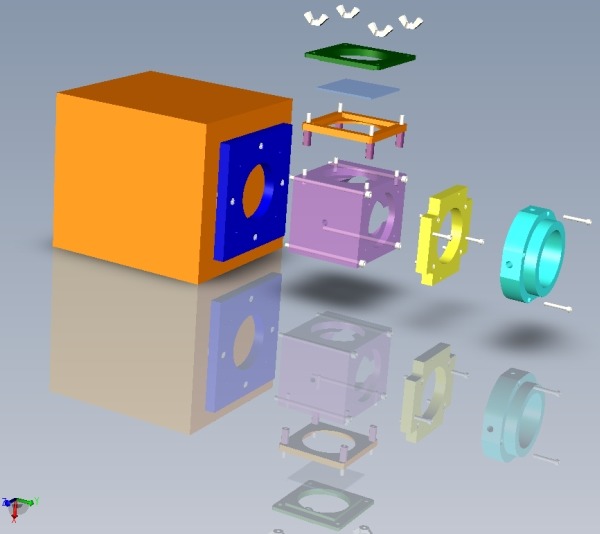
Exploded and assembled views of the mechanical design.

**Figure 4:**
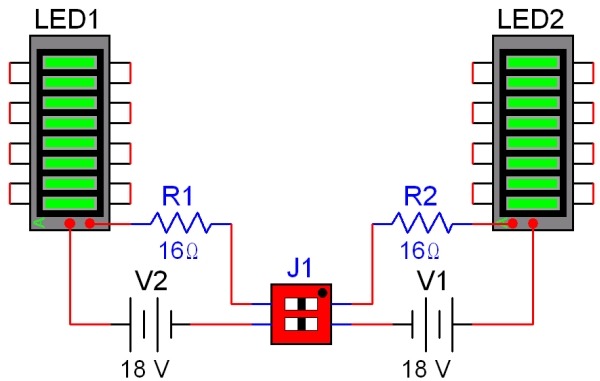
Schematic of the LED array circuit. Each LED array is powered by its own 24 V power source. The LED arrays emit green light at approximately 530 nm. The typical luminous flux is 540 ϕv at 350 m.

The mechanical design of this system involved creating interfaces for each of the aforementioned components; the components were designed with Pro/Engineer (PTC Corporation, Needham, MA, USA). An exploded view of this system is shown in Figure 3. A LED array was created to illuminate the specimen and activate the dye. The circuit diagram for the array is shown in Figure 4; the circuit was designed to power two 18 element LED arrays simultaneously with one switch and a 24 V source. The LEDs (model LXHL-MMJA, Lumiled Lighting LLC, San Jose, CA, USA) provided 530 ± 20 nm of light with a typical luminous flux of 540 ϕv at 350 mA.

An acrylic plate was created for the PDA such that it could be mounted onto a bracket. The bracket was attached to a StableRodTM vibration damped mounting post with a permanent base (model 07DPR012, Melles Griot, Carlsbad, CA, USA). The geared post allowed for smooth vertical movement of the PDA in a 300 mm range. This feature allowed for preparations of various dimensions to be studied. The base was mounted to a vibration resistant workstation with an air suspension surface to reduce motion artifact due to vibration (model 9100, Kinetic Systems Inc, Boston, MA, USA).

### Software

#### Virtual instrument

The data acquisition application with graphical user interface was programmed with LabView (National Instruments). The virtual instrument (vi) allowed for simultaneous acquisition of all 256 channels. The sampling rate was adjustable, but was set for the described studies at 1,000 samples/second.

**Figure 5:**
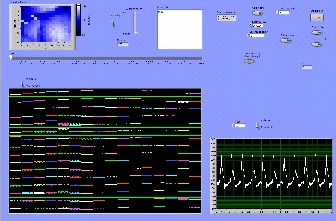
The graphical user interface created in LabView. Each photodiode output is shown in an intensity chart and waveform graph in the same sequence as the arrangement of the diodes in the photodiode array. The signal is shown with 1 ms time increments and 16 bit ADC units.

The vi displayed the acquired signal for 1-5 seconds (adjustable) and presented it in x-y chart format as well as via an intensity graph. A screenshot of the vi output is shown in Figure 5. The vi also allowed the user to save data from each individual channel for later analysis. This application also allowed the user to replay saved data files. Furthermore, to ensure data acquisition was performed under steady-state conditions, the vi had a timer with an audible alert that delayed data acquisition for a preset amount of time. This timer allowed the user to turn on the excitation lights just before acquisition in order to minimize extraneous light exposure and minimize photo bleaching.

A Matlab (Mathworks, Natick, MA, USA) application was created to visualize the wavefront of propagation (for each of the 256 signals) over time. Further information about the Matlab interface can be found in a separate publication [9].

#### Digital filter

**Figure 6:**
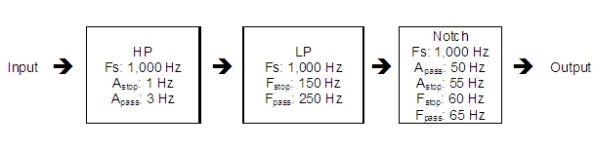
Optical mapping system filter cascade. HP=high pass filter; LP=low pass filter.

A cascaded filter approach was used to post process the acquired 256 element array sampled at 1,000 Hz. Spectral density measurements using the Welch method revealed major peaks occurring at 60 Hz and associated harmonic frequencies. To reduce this noise, the following filter cascade was implemented in Matlab, as shown in Figure 6.

The high pass filter (HP) was designed to have a sharp roll (high order), such that low frequency noise was eliminated while preserving as much of the signal as possible above 3 Hz. The low pass filter (LP) was designed to have a slow roll (low order) for the same reasons. The notch filter was intentionally designed to be narrow in bandwidth to preserve frequency content while eliminating 50/60 Hz AC coupled noise.

## Results

**Figure 7:**
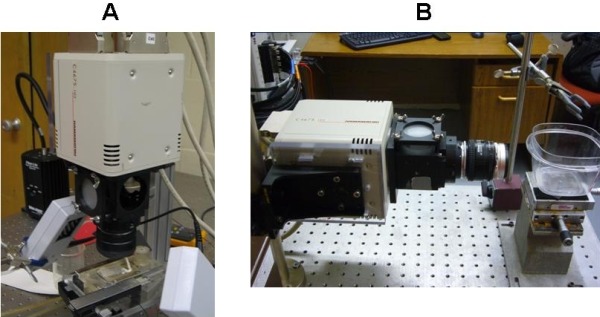
The optical mapping system is configurable for vertical mount (A) or horizontal mount (B) orientation depending on the tissue preparation of interest.

The assembled PDA unit with the compound lens, camera lens mount, cube fixture, projection screen, emission filters, PDA-to-cube interface panel, tissue preparation chamber, and LED excitation lights is shown in Figure 7. The attachment of the lens to the cube and PDA aided in providing a precise, consistent alignment between the PDA elements and the tissue preparation. A 3-axis micropositioner mounted on the heart chamber was used to position the epicardial surface of the heart in the optical field of view. Small adjustments in the x-y direction were handled by fine adjustment knobs on the tissue bath apparatus. In addition, minute focal length adjustments were easily made by the gear driven height adjustment mechanism.

The analysis of results was facilitated by the interactive and user-friendly graphical user interface, as shown in Figure 5. The intensity chart and playback feature allowed for visualization of the propagation of the action potential on a frame-by-frame basis with 1 ms resolution (default sampling rate of 1,000 Hz). The simultaneous view of the two-dimensional graphs permitted inspection of the action potential signal as a function of time.

**Figure 8:**
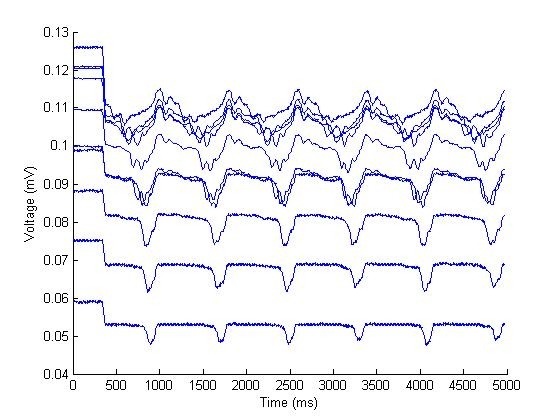
Cardiac action potential captured via mapping system. The step before 500 ms demonstrates the voltage baseline without LED illumination.

**Figure 9:**
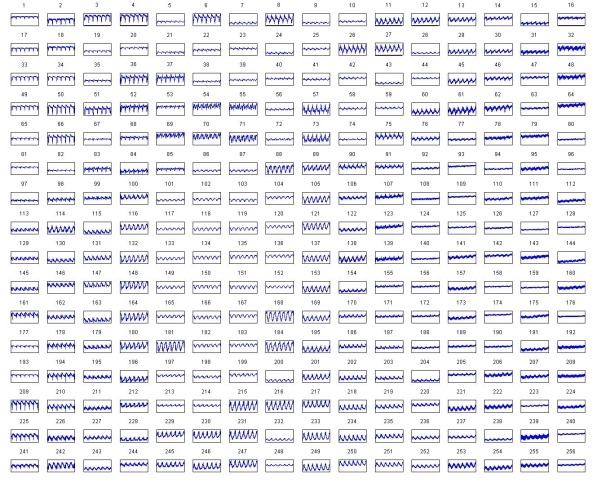
Normal sinus rhythm as shown from all 256 channels.

Figure 8 displays optical mapping signals from normal sinus rhythm. The LabView virtual interface also had a playback mode which allowed for viewing the captured frames as a moving image. Figure 9 displays results from all 256 channels.

## Discussion

### System evaluation

The high-speed data acquisition system developed and employed here has a sampling rate of 250 kilosamples per second over 80 channels, or a maximum sampling rate of 3,125 samples per second per channel; this rate could be sustained for several seconds [personal communication with National Instruments, May 23, 2005]. Other studies in our lab indicate that the typical rise time for maximum upstroke (phase 1) of the action potential is 7-10 ms in left ventricular muscle in swine (in situ) and 6-10 ms in human muscle (in vitro) [10]. This upstroke rate was slightly faster (15-20%) in ischemic swine models [11,12]. At the maximum setting, the system has the capability to capture signals with approximately 0.3 ms of resolution, which would be adequate to sample signals in both normal and ischemic (slower dynamic) conditions. It should be noted that it has been reported that a bandwidth between 0.05-1,000 Hz is generally accepted for monophasic action potential recordings in humans [13].

The need to image at high spatial resolutions and high speeds across many channels is critical to the system's data throughput. Data throughput is typically calculated as the product of temporal and spatial resolution, in units of Mbytes/s (MBs) [3]. The data throughput of the Hamamatsu PDA is 0.6 MBs with a temporal resolution of 1,000 frames per second. Here the PXI-based DAQ boards were interfaced with the PCI backplane computer by a high-speed copper data link capable of 1.5 Gbits per second serial data transfer rate. The computer's PCI express slot is capable of high bandwidth data throughput, from 5-80 Gbits per second (depending on implementation) [14]. Our computer system used a SATA standardized protocol to transfer data to a hard disk; this data throughput rate ranges from 130-150 MBs per second. These figures indicate that such a system should have adequate data flow characteristics to image cardiac action potentials, convert these analog signals to 16 bit digital words, and store the information to a local hard disk without complications due to the lack of throughput. This computer system could be easily upgraded as such technologies continue to advance.

### Unique features of the current design

This system entails two unique features that may distinguish it from other similar PDA-based equipment that has used di-4-ANEPPS for experimentation. One feature is the lack of additional hardware for signal filtering; many systems employ one or two amplification stages to achieve the desired signal amplitudes [15]. The optical mapping scheme described here relies on well shielded and grounded equipment that reduces electromagnetic interference and noise. Additionally, the lowest possible voltage range with the DAQs was used here (±200 mV) provides 112 μV of accuracy and 5.2 μV of sensitivity over 16 bits of precision.

The other distinguishing characteristic of this system is the use of LED arrays. Many systems currently employ high-intensity xenon lamps, mercury-xenon lamps, or high wattage quartz-tungsten halogen lamps. Such lamps, though versatile, have several drawbacks when compared to LED arrays, including: significant costs, additional safety measures (due to the high operating voltages and emission of ultraviolet radiation), and potential deleterious effects on measured signals due to photo-bleaching effects.

The LED arrays also require less peripheral light filtering equipment. The green LEDs used in this setup provide intensity maxima consistent with the excitation spectra for di-4-ANEPPS [6,7]. Thus, the use of LED arrays reduces the need for excitation filters which are required with broad spectrum lamps.

Finally, this system is constructed with "off the shelf" components. The total system costs (<$20,000 USD) are listed in Table 1. Depending on system configuration, commercial systems can cost several times more than this and still require extensive delivery times and service agreements [16].

### Applications of optical mapping

Fast fluorescent probe-based mapping of cardiac electrical activity has played an important role in the investigation of mechanisms of arrhythmias. More specifically, optical mapping has been particularly important when employed for preparations in which the structural complexity makes it difficult to use conventional contact electrode mapping techniques, such as in nodal areas [2,10]. Additionally, optical mapping techniques have been used to study underlying mechanisms and site origins of ventricular fibrillation. For example, a healthy atrioventricular node (AVN) plays an important role in the protection of the ventricles during tachycardia. It also serves as the pacemaker in the event of sinoatrial node (SAN) failure. An investigation of the structure-function relationship of this node through mapping can facilitate understanding of the mechanisms underlying reentrant circuits during AVN tachycardia [17,18]. More specifically, optical mapping techniques have shown phase singularity point, wave breaks, and rotor formation in humans which has helped our understanding of the mechanisms of ventricular fibrillation and defibrillation in humans [19]. Optical mapping techniques have also been used to image ventricular fibrillation from the entire ventricular surface of swine hearts, where researchers were able to identify and track ventricular fibrillation wavefront and phase singularities and to introduce the notion that rotor's central phase singularity can change [20]. Rabbit SAN activation patterns under the influence of cholinergic and adrenergic factors have been studied by recording beat-to-beat changes with an optical mapping system, where it was found that these factors cause pacemaker shifts in the rabbit SAN [21]. These techniques have enabled recording of ventricular fibrillation that has been useful in studying underlying mechanisms of sudden cardiac death. For example, polarization abnormalities were recorded using an optical mapping system to study their impact on ventricular fibrillation in Brugada Syndrome, a genetic mutation that has been linked to tachycardia and subsequent sudden cardiac death [22,23]. Brugada Syndrome manifests itself in three different ways via ECG: 1) Slight ST and J point elevation and negative T wave 2) ST and J point elevation with biphasic T wave, and 3) similar to type I but with less elevation in fiducials. Optical mapping has also allowed researchers to discover that injured cardiomyocytes can re-establish electrical conduction with regenerating cardiomyocytes between 2-4 weeks after injury by mapping the myocardium in ventricles of zebrafish. These findings have shown potential for regeneration in injured human hearts [24]. We plan to use this system for future investigation of isolated human trabeculae obtained from either explanted hearts or from organ donors whose hearts are not deemed viable for transplant.

It should be noted that the use of optical mapping systems is not limited to the recording of transmembrane potentials. Other fluorescent indicators of important cellular parameters, such as intracellular calcium and potassium, can be used as well [6]. Intracellular calcium has many effects on the electrophysiology of the heart including a key role in electrical alternans [25], heart failure [26,27], and ventricular fibrillation [28]. The emission spectra of di-4-ANEPPS and the calcium stain indo-1 (excitation at 365 nm) can be used to simultaneously measure action potentials and calcium transients from heart preparations and intact perfused hearts. For example, optical mapping was used to map ventricular arrhythmias caused by RYR2 mutations. These mutations can lead to catecholaminergic polymorphic ventricular tachycardia, bradycardia, and subsequent sudden cardiac death. These techniques were able to show that in hearts with RYR2 mutations, arrhythmias can be caused by a catecholaminergic and/or intracellular Ca^2+^ overload-dependent mechanism [29]. The slowing of the heart rate is in part affected by acetylcholine-dependent activation of the K^+^ channel I_KACh_ [30]. Optical mapping techniques have been used to study the changes in electrical activity due to these cholinergic changes in atrium preparations of fish, amphibian, and reptilian models. Research has indicated that cholinergic suppression of electrical activity in the atria of these animals is common and is caused by I_KACh_ activation. These findings indicate that I_KACh_ channels may be important for initiation of atrial arrhythmias [31].

## Conclusion

Optical mapping provides specific opportunities to explore clinically important phenomena related to cellular function. The experimental setup presented here demonstrates a high-resolution, high-speed optical mapping system for the study of cardiac action potentials on a micro or macro scale. The modular design of our system allows unique flexibility to study either cellular or tissue preparations, i.e., by employing a vertically mounted PDA as well as whole heart preparations with a rail mounted horizontal system. The system also incorporates custom mechanical fixtures that make it easy to align the center of the PDA element grid, the focal point of the lens, and the projection screen regardless of the system's mounting. The high-speed data acquisition boards provide suitable bandwidth for streamlined data flow and the data acquisition software allows recording and analyses of high spatial and temporal resolution cardiac action potentials. Finally, our system has capabilities to expand its use and application to any area that requires measurement of physiological signals of interest based on fluorescent probes.

## Figures and Tables

**Table 1:**
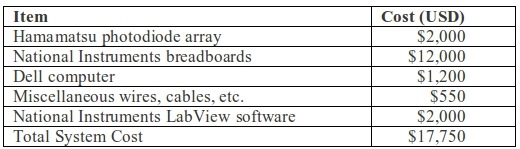
Optical mapping gross bill of materials and associated costs
